# Association between NADPH Oxidase p22^phox^ C242T Polymorphism and Ischemic Cerebrovascular Disease: A Meta-Analysis

**DOI:** 10.1371/journal.pone.0056478

**Published:** 2013-02-11

**Authors:** Bing-Hu Li, Li-Li Zhang, Bei-Bei Zhang, Yan-Wei Yin, Li-Meng Dai, Yan Pi, Lu Guo, Chang-Yue Gao, Chuan-Qin Fang, Jing-Zhou Wang, Jing-Cheng Li

**Affiliations:** 1 Department of Neurology, Institute of Surgery Research, Daping Hospital, Third Military Medical University, Yuzhong District, Chongqing, PR China; 2 Department of Medical Affairs, General Hospital of PLA Chengdu Military Area Command, Chengdu, PR China; 3 Department of Medical Genetics, College of Basic Medical Science, Third Military Medical University, Shapingba District, Chongqing, PR China; National University of Ireland Galway, Ireland

## Abstract

**Background:**

Epidemiological studies have evaluated the association between nicotinamide adenine dinucleotide phosphate (NADPH) oxidase p22^phox^ C242T polymorphism and risk of ischemic cerebrovascular disease (ICVD), but the results remain inconclusive. This meta-analysis was therefore designed to clarify these controversies.

**Methodology/Principal Findings:**

Systematic searches of electronic databases Embase, PubMed and Web of Science, as well as hand searching of the references of identified articles and the meeting abstracts were performed. Statistical analyses were performed using software Review Manager (Version 5.1.7) and Stata (Version 11.0). The pooled odds ratios (ORs) with 95% confidence intervals (95%CIs) were performed. Fixed or random effects model was separately used depending on the heterogeneity between studies. Publication bias was tested by Begg's funnel plot and Egger's regression test. A total of 6 studies including 1,948 cases and 2,357 controls were combined showing no statistical evidence of association between NADPH oxidase p22^phox^ C242T polymorphism and overall ICVD (allelic model: OR = 1.08, 95%CI = 0.93–1.26; additive model: OR = 1.33, 95%CI = 0.81–2.17; dominant model: OR = 1.00, 95%CI = 0.86–1.15; recessive model: OR = 1.06, 95%CI = 0.77–1.45). Significant association was found in large-artery atherosclerotic stroke subgroup (allelic model: OR = 1.12, 95%CI = 0.88–1.41; additive model: OR = 1.36, 95%CI = 0.60–3.09; dominant model: OR = 1.25, 95%CI = 0.74–2.11; recessive model: OR = 2.17, 95%CI = 1.11–4.23). No statistical evidence of significant association was observed for small-vessel occlusive stroke, as well as Asian subgroup and Caucasian subgroup. Statistical powers on the combined sample size (total and subgroup) were all lower than 80%.

**Conclusions/Significance:**

This meta-analysis indicates that NADPH oxidase p22^phox^ C242T polymorphism is more associated with large-artery atherosclerotic stroke than small-vessel occlusive stroke. However, this conclusion should be interpreted with caution due to the small sample size. Larger sample-size studies with homogeneous ICVD patients and well-matched controls are required.

## Introduction

Reactive oxygen species (ROS) has been suggested to play a major role in vascular disease [1]–[7]. The most significant sources of ROS in the vascular system are nicotinamide adenine dinucleotide phosphate (NADPH) oxidases [8], which include two membrane-bound subunits Nox2 and p22^phox^ and the cytosolic components p47^phox^, p67^phox^, p40^phox^ and Rac-1 [9], [10]. The p22^phox^ subunit, which binding to Nox proteins leads to protein stabilization, is essential for the activation of NADPH oxidase [11]–[13]. The p22^phox^ is encoded by the CYBA gene, which is located on the long arm of chromosome 16 at position 24 [14]. Several polymorphisms of the CYBA gene have been reported, which could lead to significant functional variation among individuals in oxidative stress by influencing gene expression and NADPH oxidase activation [15]–[17]. Among them, the C242T polymorphism, which is located in exon 4 at position 214 from the ATG codon, is a well studied one. The C242T polymorphism, resulting from functional C-to-T substitution, has been reported to go along with a reduction in the generation of superoxide anions in the vascular wall [18] and closely with various diseases including renal disease [19], hypertension [20], diabetes [21], cardiovascular disease [22], [23] and cerebrovascular disease [24], [25]. As for ischemic cerebrovascular disease (ICVD), a variety of epidemiological studies have evaluated the role of NADPH oxidase p22^phox^ C242T polymorphism, but the results were inconclusive [24], [26]–[30]. It is likely that NADPH oxidase p22^phox^ C242T polymorphism may influence the susceptibility of ICVD. The present meta-analysis was therefore designed to derive a more precise estimation of the association between NADPH oxidase p22^phox^ C242T polymorphism and ICVD.

## Methods and Materials

### Data sources

This meta-analysis followed the Preferred Reporting Items for Systematic Reviews and Meta-analyses (PRISMA) criteria [31] and Meta-analysis of Observational Studies in Epidemiology (MOOSE) guidelines [32]. We selected possibly relevant articles in Embase (1966–June 2012), PubMed (up to June 2012) and Web of Science (1950–June 2012) (last search was update on June 1, 2012) with search strategy: “NADPH oxidase” AND “mutation OR variant OR polymorphism OR genotype” AND “stroke OR cerebrovascular disease OR cerebrovascular disorder OR cerebral infarction OR cerebral ischemia OR brain infarction”. Other relevant studies were identified by hand-searching the references of included articles identified by electronic search and the abstracts presented at related scientific societies meetings. The search was limited to English and Chinese language papers and human subject studies. Two investigators (Li BH and Zhang LL) screened each of the titles, abstracts and full texts to determine inclusion independently. The results were compared and disagreements were resolved by consensus. Detailed search strategy is available at the **Appendix S1**.

### Inclusion criteria

Studies were included in the meta-analysis if: (1) Studies on the relationship between NADPH oxidase C242T polymorphism and ICVD; (2) ICVD includes ischemic stroke and transient ischemic attack; (3) Published case-control, nested case-control or cohort designs studies; (4) Studies with full text articles; (5) Studies reporting odds ratios (ORs) with 95% confidence intervals (CIs) or raw data for their calculations. Studies deviating from Hardy-Weinberg equilibrium (HWE) were not removed.

### Data extraction

Information was carefully extracted from all included publications independently by two of the authors (Li BH and Zhang LL) according to the inclusion criteria listed above. Disagreement was resolved by consensus. If these two authors could not reach a consensus, another author (Li JC) was consulted. The following data were collected from each study: first author's name, publication date, country, ethnicity, study design (source of controls), phenotype (type of ICVD), diagnoses of ICVD (clinical or imaging diagnosis), total number of cases and controls, frequency of C242T polymorphism in cases and controls or published crude ORs derived from these data and evidence of HWE (*P* value less than 0.05 of HWE was considered significant), respectively. Different ethnicities were categorized as Caucasian, Asian, African and mixed. Study design was stratified to population-based (PB) studies and hospital-based (HB) studies. According to the TOAST (Trial of Org 10172 in Acute Stroke Treatment) classification, there are five subtypes of ischemic stroke: large-artery atherosclerosis, cardioembolism, small-vessel occlusion, stroke of other determined etiology, and stroke of undetermined etiology [33]. When studies reported genotype distributions for ischemic stroke subtype, we also extracted data of each subtype separately for subgroup analyses. If necessary data were not reported in the primary manuscripts, we contacted the corresponding authors by email to request the missing data.

### Quality score assessment

The qualities of included studies were assessed independently by the same two investigators using the Newcastle-Ottawa Scale (NOS) [34]. The NOS uses a ‘star’ rating system to judge quality based on 3 aspects of the study: selection, comparability, and exposure (case-control studies) or outcome (cohort studies). Scores were ranged from 0 star (worst) to 9 stars (best). Studies with a score ≥7 were considered to be of high quality. Disagreement was settled as described above.

### Statistical analysis

The strength of association between C242T polymorphism and ICVD risk was measured by ORs and 95% CIs. The combined ORs and 95% CIs were calculated respectively for allelic model (T vs. C), additive model (TT vs. CC), dominant model (TT+TC vs. CC) and recessive model (TT vs. TC+CC) [35]. We used the generic inverse variance method to obtain pooled ORs and 95% CIs, weighting each study by the inverse of the square of the standard error of its study-specific OR. We only used the crude ORs and 95%CIs for meta-analysis. If the studies did not provide crude ORs and 95%CIs, we calculated the ORs and 95%CIs by the total number of cases and controls, and frequency of C242T polymorphism in cases and controls. A fixed effects model was adopted when no heterogeneity was observed among studies. Otherwise, a random effects model was adopted. Between-study heterogeneity was assessed by the Q-test and I^2^ statistic, *P*<0.10 and I^2^>50% indicated evidence of heterogeneity [36], [37]. Subgroup analyses were performed by ischemic stroke subtypes. Sensitivity analysis was performed by including studies of one ethnic group and by limiting the meta-analysis to studies in agreement with HWE to identify if different results were seen. Publication bias was examined by plotting a Begg's funnel plot and Egger's regression test (*P*<0.05 was considered representative of statistically significant publication bias) [38]. All above statistical analyses were performed using Review Manager 5.1.7 and Stata 11.0. Power analysis was performed using Quanto software package (Version 1.2.4, http://hydra.usc.edu/gxe/) [39]. We assumed an unmatched case-control design and considered a two-sided p-value of 0.05.

## Results

### Study characteristics

**Figure 1:**
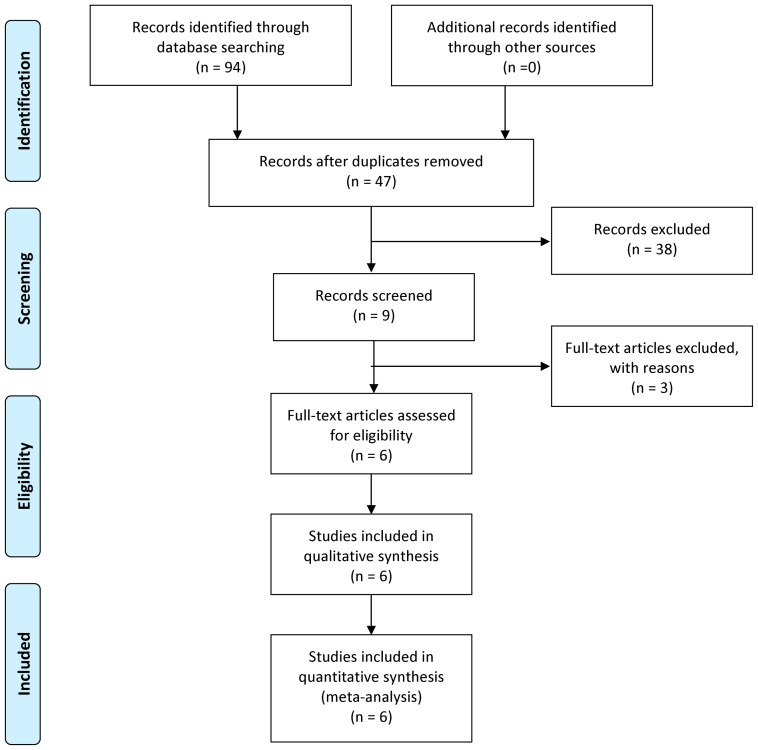
Flow diagram of the selection of eligible studies.

The present study met the PRISMA statement requirements (**Appendix S2**) and MOOSE guidelines (**Appendix S3**). The study selection process is detailed in **Figure 1**. Based on our preliminary search criteria, a total of nine publications were eligible [24], [26]–[30], [40]–[42]. Among these articles, one study was review article [40]. Two studies reported the p47^phox^ C923T rather than p22^phox^ C242T polymorphism [41], [42]. Hence, six studies were included in the final meta-analysis, including 1,948 cases and 2,357 controls. Khan et al. provided no frequency of C242T polymorphism in cases and controls, and we failed to obtain these data by contacting the corresponding author, which limited our calculation of OR and 95%CI for allelic model and additive model. All studies were case-control in design. **Table 1** shows the studies identified and their main characteristics. The NOS results showed that the average score was 8.5 (range 8 to 9), indicating that the methodological quality was generally good. Statistical powers based on the given sample size of each study ranged from 5.1% to 70.8%, which were all lower than 80%. Among the six articles, three focused on Asians, and three on Europeans. The countries of these studies included Japan, UK, Germany and Poland. Five studies characterized ischemic stroke subtypes in their analyses, allowing subtype specific meta-analysis. However, because of the limited available data on cardioembolic, other determined etiology and undetermined subtypes, we only made meta-analysis for small-vessel occlusive ischemic stroke and large-artery atherosclerotic ischemic stroke, in which five studies were combined for small-vessel occlusive subtype [24], [26]–[29] and four for large-artery atherosclerotic subtype [24], [26]–[28].

**Table 1:** Main characteristics of studies included in the meta-analysis.

First author	Year	Country	Ethnicity	SOC	Genotypes distribution case/control, N(%)			MAF case/control, N(%)	Subtype of ICVD (diagnostic methods)	Adjustment for confounders	HWE Y/N(P)	Score	Sample size (power)
					CC	TC	TT	T					
Ito	2000	Japan	Asian	PB	177(78.3)/261(86.7)	46(20.4)/38(12.6)	3(1.3)/2(0.7)	52(11.5)/42(7.0)	SVO, LAA and TIA (brain CT and/or MRI)	age and sex matched	Y(0.63)	8	226/301 (70.8%)
Shimo-Nakanishi	2004	Japan	Asian	PB	102(85.0)/154(87.0)	18(15.0)/23(13.0)	0(0.0)/0(0.0)	18(7.5)/23(6.5)	SVO and LAA (brain CT and/or MRI)	age, sex and ethnicity matched	Y(0.36)	9	120/177 (47.5%)
Kuroda	2007	Japan	Asian	PB	851(80.7)/840(79.6)	189(17.9)/198(18.8)	15(1.4)/17(1.6)	219(10.4)/232(11.0)	SVO,LAA, CE and SUE (brain CT and/or MRI)	age, sex and ethnicity matched	Y(0.18)	9	1055/1055 (9.5%)
Khan	2007	UK	Caucasian	PB	-	-	-	-	SVO (brain CT and/or MRI)	age, sex and ethnicity matched	NE	9	316/638 (19.21%)
Genius	2008	Germany	Caucasian	PB	73(45.3)/62(45.6)	66(41.0)/68(50.0)	22(13.7)/6(4.4)	110(34.2)/80(29.4)	SVO,LAA, CE, SUE and TIA (brain CT and/or MRI)	sex and ethnicity matched	N(0.02)	8	161/136 (27.2%)
Niemiec	2010	Poland	Caucasian	PB	35(50.0)/26(52.0)	27(38.6)/18(36.0)	8(11.4)/6(12.0)	43(30.7)/30(30.0)	Total ICVD (neuroimaging)	age and sex matched	Y(0.31)	8	70/50 (5.1%)

SOC: source of controls; PB: population-based.

MAF: minor allele frequency.

HWE: Hardy-Weinberg equilibrium; Y: yes; N: no; NE: not estimate.

TIA: transient ischemic attack; ICVD: ischemic cerebrovascular disease.

SVO: small-vessel occlusion; LAA: large-artery atherosclerosis; CE: cardioembolism; SUE: stroke of undetermined etiology.

CT: computed tomography; MRI: magnetic resonance imaging.

### Quantitative synthesis

**Figure 2:**
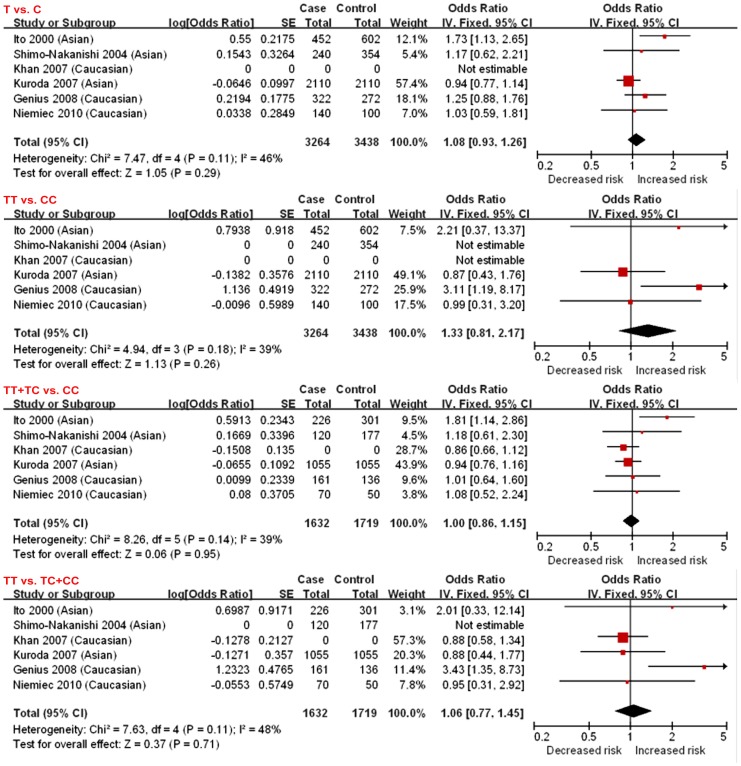
Forest plots for overall studies. Fixed effects models were used as no statistical heterogeneity across studies were observed (I^2^ = 46%, p = 0.11; I^2^ = 39%, p = 0.18; I^2^ = 39%, p = 0.14; I^2^ = 48%, p = 0.11, respectively). There was no statistical evidence of association (T vs. C: OR = 1.08, 95%CI = 0.93–1.26; TT vs. CC: OR = 1.33, 95%CI = 0.81–2.17; TT+TC vs. CC: OR = 1.00, 95%CI = 0.86–1.15; and TT vs. TC+CC: OR = 1.06, 95%CI = 0.77–1.45). se: standard error; IV: inverse variance; CI: confidence interval.

Fixed effect models were adopted as no heterogeneity was observed among studies for all the genetic models (I^2^ = 46%, *P* = 0.11; I^2^ = 39%, *P* = 0.18; I^2^ = 39%, *P* = 0.14; I^2^ = 48%, *P* = 0.11, respectively). When all six studies including 1,948 cases and 2,357 controls were pooled into the meta-analysis, there was no statistical evidence of association between p22^phox^ C242T polymorphism and overall ICVD (allelic model: OR = 1.08, 95%CI = 0.93–1.26; additive model: OR = 1.33, 95%CI = 0.81–2.17; dominant model: OR = 1.00, 95%CI = 0.86–1.15; and recessive model: OR = 1.06, 95%CI = 0.77–1.45) (**Figure 2**). Power calculation on the pooled sample size showed that the statistical power was 21.4%, which was lower than 80%.

Four studies including 526 cases and 1,669 controls were pooled into the meta-analysis for large-artery atherosclerotic subtype. Statistically significant association was found in recessive model (allelic model: OR = 1.12, 95%CI = 0.88–1.41; additive model: OR = 1.36, 95%CI = 0.60–3.09; dominant model: OR = 1.25, 95%CI = 0.74–2.11; and recessive model: OR = 2.17, 95%CI = 1.11–4.23) (**Appendix S4**). Power calculation on the combined sample size showed that the statistical power was 40.8%, which was lower than 80%. Five studies including 959 cases and 2,307 controls were pooled into the meta-analysis for small-vessel occlusive subtype. There was no statistical evidence of significant association (allelic model: OR = 1.16, 95%CI = 0.94–1.44; additive model: OR = 1.32, 95%CI = 0.28–6.12; dominant model: OR = 1.11, 95%CI = 0.84–1.47; and recessive model: OR = 0.90, 95%CI = 0.62–1.31) (**Appendix S5**). Power calculation on the pooled sample size showed that the statistical power was 38.6%, which was lower than 80%.

### Sensitivity analysis

The combined minor allele frequency (MAF) was 33.1% for Caucasian cases and 10.3% for Asian cases. Considering ethnic variations, sensitivity analysis was firstly performed by including studies of one ethnic group. Three studies including 547 cases and 824 controls were pooled into the meta-analysis for Caucasian subgroup. The combined ORs and 95%CIs were: for allelic model: OR = 1.18, 95%CI = 0.88–1.59; for additive model: OR = 1.85, 95%CI = 0.60–5.65; for dominant model: OR = 0.91, 95%CI = 0.73–1.13; and for recessive model: OR = 1.36, 95%CI = 0.57–3.22 (**Appendix S6**). Power calculation on the pooled sample size showed that the statistical power was 50.3%, which was lower than 80%. Three studies including 1,401 cases and 1,533 controls were pooled into the meta-analysis for Asian subgroup. The combined ORs and 95%CIs were: for allelic model: OR = 1.20, 95%CI = 0.79–1.84; for additive model: OR = 0.98, 95%CI = 0.51–1.89; for dominant model: OR = 1.22, 95%CI = 0.78–1.91; and for recessive model: OR = 0.98, 95%CI = 0.51–1.88 (**Appendix S7**). Power calculation on the pooled sample size showed that the statistical power was 57.0%, which was lower than 80%.

Secondly, we performed sensitivity analysis by limiting the meta-analysis to studies in agreement with HWE. One study [27] deviating from HWE was excluded. The corresponding pooled ORs and 95%CIs were not substantively altered (allelic model: OR = 1.15, 95%CI = 0.84–1.58; additive model: OR = 0.99, 95%CI = 0.56–1.74; dominant model: OR = 1.07, 95%CI = 0.83–1.37; and recessive model: OR = 0.91, 95%CI = 0.65–1.27), indicating that our results were robust.

### Publication bias

**Figure 3:**
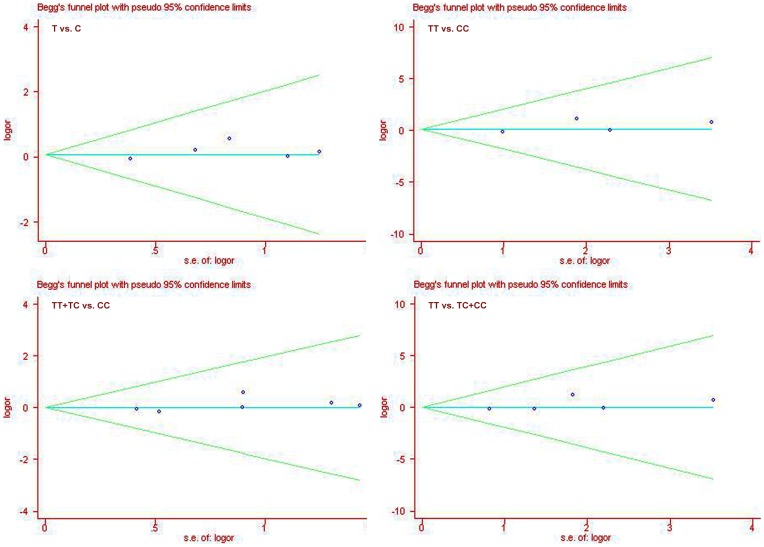
Funnel plots for overall studies. The shapes of the funnel plots did not reveal any evidence of obvious asymmetry visually. se: standard error; OR: odds ratio.

The shapes of the funnel plots did not reveal any evidence of obvious asymmetry visually (**Figure 3**). Also there was no statistical evidence of publication bias among studies by using Egger's regression test (*P* = 0.28 for allelic model; *P* = 0.38 for additive model; *P* = 0.24 for dominant model; and *P* = 0.32 for recessive model, respectively).

## Discussion

ICVD is a multifactorial disease, leading to a high mortality and disability rate [43]. It is well known that stroke is associated closely with conventional vascular risk factors [44] and genetic factors. Increasing evidences from evidence-based studies support the critical role of genetic factors in the development of ICVD [45], [46]. Recently, a variety of studies have focused on the association between NADPH oxidase p22^phox^ C242T mutation and ICVD. However, the observed associations of these studies were inconclusive and a single study may be too underpowered to detect a possible small effect of the gene polymorphism on ICVD, especially when the sample size is relatively small. Meta-analysis has the benefit to overcome this limitation by increasing the sample size and may generate more precise results, which has been widely used in genetic association studies [47], [48]. The present meta-analysis was therefore carried out.

Based on our study selection process, a total of six publications were included in our meta-analysis. Ito et al. reported that the C242T polymorphism was a novel pathogenetic risk factor for ICVD with a fact that TC+TT genotype was significantly higher in the ICVD patients [24], which was confirmed by Genius et al., demonstrating that homozygosity for the T variant was associated with an enhanced risk for cerebral ischemia [27]. Our meta-analyses did not show statistical evidence of association between the NADPH oxidase p22^phox^ C242T polymorphism and ICVD in the overall study population. This finding is consistent with most of the included studies.

Theoretically, an impaired NADPH oxidase activity was linked to procoagulant state [49] and hypercholesterolemia [50] and thus increase the risk of ICVD. However, a negative result is reached. Possible explanation may be that: (1) The combined sample sizes in our study were still inadequate to detect the association between C242T polymorphism and ICVD because power calculations for the combined sample size demonstrated that all the meta-analyses were underpowered; (2) Other enzyme systems such as the xanthine oxidase also contributes to the generation of ROS [51]. The C242T polymorphism on ICVD might be confounded by the genetic variation in these systems, so further studies were required; (3) The C242T polymorphism may be associated with the specific subtypes of ischemic stroke as some studies showed that genetic factors were more associated with small- and large-vessel stroke than cardioembolic stroke [52], [53]. Regarding C242T polymorphism, Ito et al. reported that the TC+TT genotype was higher in the ICVD patients with atherothrombotic infarction than those with lacunar infarction and transient ischemic attack. We also analyzed this potential association in our study based on available data. Results showed that C242T polymorphism was more associated with large-artery atherosclerotic stroke than small-vessel occlusive stroke. In the subgroup analysis of large-artery atherosclerotic stroke, statistically significant association was found in recessive model, in which the OR was 2.17. It indicated that TT genotype could increase the risk of large-artery atherosclerotic stroke of 2.17 fold, suggesting that individual with homozygous TT genotype could have higher risk of large-artery atherosclerotic stroke than CC and TC genotype. However, based on the combined sample size (526 cases and 1,669 controls for large-artery atherosclerotic subtype and 959 cases and 2,307 controls for small-vessel occlusive subtype), power calculation showed that it was underpowered to detect the association between C242T polymorphism and both subtype.

With respect to MAF of the p22^phox^ C242T, it is quite different between Caucasian population and Asian population. Data of International HapMap Project shows that the MAF of the p22^phox^ C242T polymorphism is common among Utah residents with Northern and Western European ancestry (CEU) (MAF = 0.314), whereas it is very low among Han Chinese in Beijing, China (CHB) and Japanese in Tokyo, Japan (JPT) (MAF = 0.084 and 0.062, respectively) [54]. Our study also showed that the combined MAF was 33.1% for Caucasian cases and 10.3% for Asian cases. Considering ethnic variations, sensitivity analysis was performed. However, both subgroups revealed no statistical evidence of significant association between p22^phox^ C242T polymorphism and ICVD.

For better interpreting the results, some limitations of this meta-analysis should be acknowledged. Firstly, the small number of studies and sample size limited the ability to draw more solid conclusions. Secondly, lacking of the original data limited our further evaluation of potential interactions among gene-gene and gene-environment.

Nonetheless, to the best of our knowledge, the present study is the first meta-analysis of the relationship between C242T polymorphism and ICVD. Our meta-analysis suggests that C242T polymorphism is more associated with large-artery atherosclerotic stroke than small-vessel occlusive stroke. However, this conclusion should be interpreted with caution due to the small sample size. Larger sample-size studies with homogeneous ICVD patients and well-matched controls are required.

## Supporting Information

Appendix S1 **Search Strategy.**
(DOC)

Appendix S2 **PRISMA 2009 Checklist.**
(DOC)

Appendix S3 **MOOSE Checklist.**
(DOC)

Appendix S4 **Forest plots for large-artery atherosclerotic subtype.**
(TIF)

Appendix S5 **Forest plots for small-vessel occlusive subtype.**
(TIF)

Appendix S6 **Forest plots for Caucasian subgroup.**
(TIF)

Appendix S7 **Forest plots for Asian subgroup.**
(TIF)
