# Supplementary material for: Association between NADPH Oxidase p22phox C242T Polymorphism and Ischemic Cerebrovascular Disease: A Meta-Analysis
Source: PLoS One. 2013 Feb 11;8(2):e56478. doi: 10.1371/journal.pone.0056478 (PMC3569432; doi:10.1371/journal.pone.0056478)
Supplement: Appendix S1 — Search Strategy. (DOC) [file pone.0056478.s001.doc]

**Appendix 1 Search strategy**

**1. PubMed search strategy** (n=47)

("nadph oxidase"[MeSH Terms] OR ("nadph"[All Fields] AND "oxidase"[All Fields]) OR "nadph oxidase"[All Fields]) OR ("nad"[MeSH Terms] OR "nad"[All Fields]) AND P[All Fields] AND (H[All Fields] AND ("oxidoreductases"[MeSH Terms] OR "oxidoreductases"[All Fields] OR "oxidase"[All Fields])) AND (("mutation"[MeSH Terms] OR "mutation"[All Fields]) OR variant[All Fields] OR ("polymorphism, genetic"[MeSH Terms] OR ("polymorphism"[All Fields] AND "genetic"[All Fields]) OR "genetic polymorphism"[All Fields] OR "polymorphism"[All Fields]) OR ("genotype"[MeSH Terms] OR "genotype"[All Fields])) AND (("stroke"[MeSH Terms] OR "stroke"[All Fields]) OR ("cerebrovascular disorders"[MeSH Terms] OR ("cerebrovascular"[All Fields] AND "disorders"[All Fields]) OR "cerebrovascular disorders"[All Fields] OR ("cerebrovascular"[All Fields] AND "disease"[All Fields]) OR "cerebrovascular disease"[All Fields]) OR ("cerebrovascular disorders"[MeSH Terms] OR ("cerebrovascular"[All Fields] AND "disorders"[All Fields]) OR "cerebrovascular disorders"[All Fields] OR ("cerebrovascular"[All Fields] AND "disorder"[All Fields]) OR "cerebrovascular disorder"[All Fields]) OR ("stroke"[MeSH Terms] OR "stroke"[All Fields] OR ("cerebral"[All Fields] AND "infarction"[All Fields]) OR "cerebral infarction"[All Fields] OR "cerebral infarction"[MeSH Terms] OR ("cerebral"[All Fields] AND "infarction"[All Fields])) OR ("cerebral ischaemia"[All Fields] OR "cerebral infarction"[MeSH Terms] OR ("cerebral"[All Fields] AND "infarction"[All Fields]) OR "cerebral infarction"[All Fields] OR ("cerebral"[All Fields] AND "ischemia"[All Fields]) OR "cerebral ischemia"[All Fields] OR "brain ischemia"[MeSH Terms] OR ("brain"[All Fields] AND "ischemia"[All Fields]) OR "brain ischemia"[All Fields] OR ("cerebral"[All Fields] AND "ischemia"[All Fields])) OR ("brain infarction"[MeSH Terms] OR ("brain"[All Fields] AND "infarction"[All Fields]) OR "brain infarction"[All Fields]))

**2. EMBASE search strategy** (n=14)

#1. 'nadph'/exp OR nadph AND ('oxidase'/exp OR oxidase)

#2. 'mutation'/exp OR mutation

#3. variant

#4. polymorphism

#5. 'genotype'/exp OR genotype

#6. #2 OR #3 OR #4 OR #5

#7. 'stroke'/exp OR stroke

#8. cerebrovascular AND ('disease'/exp OR disease)

#9. cerebrovascular AND ('disorder'/exp OR disorder)

#10. cerebral AND ('infarction'/exp OR infarction)

#11. cerebral AND ('ischemia'/exp OR ischemia)

#12. 'brain'/exp OR brain AND ('infarction'/exp OR infarction)

#13. #7 OR #8 OR #9 OR #10 OR #11 OR #12

#14. #1 AND #6 AND #13

**3. Web of Science search strategy (**n=33)

# 1 topic= (NADPH oxidase)

# 2 topic= (mutation)

# 3 topic = (variant)

# 4 topic = (polymorphism)

# 5 topic = (genotype)

# 6 #5 OR #4 OR #3 OR #2

# 7 topic = (stroke)

# 8 topic = (cerebrovascular disease)

# 9 topic = (cerebrovascular disorder)

# 10 topic = (cerebral infarction)

# 11 topic = (cerebral ischemia)

# 12 topic = (brain infarction)

# 13 #12 OR #11 OR #10 OR #9 OR #8 OR #7

# 14 #13 AND #6 AND #1

**4. No articles identified by manual search and abstracts of conferences.**
