# Supplementary material for: Association between NADPH Oxidase p22phox C242T Polymorphism and Ischemic Cerebrovascular Disease: A Meta-Analysis
Source: PLoS One. 2013 Feb 11;8(2):e56478. doi: 10.1371/journal.pone.0056478 (PMC3569432; doi:10.1371/journal.pone.0056478)
Supplement: Appendix S2 — PRISMA 2009 Checklist. (DOC) [file pone.0056478.s002.doc]

| **Section/topic** | **#** | **Checklist item** | **Page #** |
| --- | --- | --- | --- |
| **TITLE** | | |  |
| Title | 1 | Association between NADPH oxidase p22phox C242T polymorphism and ischemic cerebrovascular disease: A meta-analysis | 1 |
| **ABSTRACT** | | |  |
| Structured summary | 2 | **Background:** Epidemiological studies have evaluated the association between NADPH oxidase p22phox C242T polymorphism and risk of ischemic cerebrovascular disease (ICVD), but the results remain inconclusive. This meta-analysis was therefore designed to clarify these controversies.  **Methodology/Principal Findings:** Systematic searches of electronic databases Embase, PubMed and Web of Science, as well as hand searching of the references of identiﬁed articles and the meeting abstracts were performed. Statistical analyses were performed using software Review Manager (Version 5.1.7) and Stata (Version 11.0). The pooled odds ratios (ORs) with 95% confidence intervals (95%CIs) were performed. Fixed or random effects model was separately used depending on the heterogeneity between studies. Publication bias was tested by Begg’s funnel plot and Egger’s regression test. A total of 6 studies including 1,948 cases and 2,357 controls were combined showing no statistical evidence of association between NADPH oxidase p22phox C242T polymorphism and overall ICVD (allelic model: OR=1.08, 95%CI=0.93-1.26; additive model: OR=1.33, 95%CI=0.81-2.17; dominant model: OR=1.00, 95%CI=0.86-1.15; recessive model: OR=1.06, 95%CI=0.77-1.45). Significant association was found in large-artery atherosclerotic stroke subgroup (allelic model: OR=1.12, 95%CI=0.88-1.41; additive model: OR=1.36, 95%CI=0.60-3.09; dominant model: OR=1.25, 95%CI=0.74-2.11; recessive model: OR=2.17, 95%CI=1.11-4.23). No statistical evidence for significant association was observed for small-vessel occlusive stroke, as well as Asian subgroup and Caucasian subgroup. Statistical powers on the combined sample size (total and subgroup) were all lower than 80%.  **Conclusions/Significance:** This meta-analysis indicates that NADPH oxidase p22phox C242T polymorphism is more associated with large-artery atherosclerotic stroke than small-vessel occlusive stroke. However, this conclusion should be interpreted with caution due to the small sample size. Larger sample-size studies with homogeneous ICVD patients and well-matched controls are required. | 2 |
| **INTRODUCTION** | | |  |
| Rationale | 3 | Reactive oxygen species (ROS) has been suggested to play a major role in the vascular disease. The most significant sources of ROS in the vascular system are NADPH oxidases, which include two membrane-bound subunits Nox2 and p22phox and the cytosolic components p47phox, p67phox, p40phox, and Rac-1. The p22phox subunit, which binding to Nox proteins lead to protein stabilization, is essential for the activation of NADPH oxidase. The p22phox is encoded by the CYBA gene, which is located on the long arm of chromosome 16 at position 24. Several polymorphisms of the CYBA gene have been reported, which could lead to signiﬁcant functional variation between individuals in oxidative stress by inﬂuencing gene expression and NADPH oxidase activation. Among them, the C242T polymorphism, which is located in exon 4 at position 214 from the ATG codon is a well studied one. The C242T polymorphism, resulting from functional C-to-T substitution, has been reported to go along with a reduction in the generation of superoxide anions in the vascular wall and closely with various diseases including renal disease, hypertension, diabetes, cardiovascular disease and cerebrovascular disease. As for ischemic cerebrovascular disease (ICVD), a variety of epidemiological studies have evaluated the role of NADPH oxidase p22phox C242T polymorphism, but the results were inconclusive. It is likely that NADPH oxidase p22phox C242T polymorphism may influence the susceptibility of ICVD. | 3 |
| Objectives | 4 | The present meta-analysis was therefore designed to derive a more precise estimation of the association between NADPH oxidase p22phox C242T polymorphism and ICVD. | 3 |
|  | | |  |
| **METHODS** | | |  |
| Protocol and registration | 5 | No protocol and registration. |  |
| Eligibility criteria | 6 | (1) Studies on the relationship between NADPH oxidase C242T polymorphism and ICVD; (2) Ischemic cerebrovascular disease includes ischemic stroke and transient ischemic attack; (3) Published case-control, nested case-control or cohort designs studies; (4) Studies with full text articles; (5) Studies reporting odds ratios (ORs) with 95% confidence intervals (CIs) or raw data for their calculation. Studies deviating from Hardy-Weinberg equilibrium (HWE) were not removed. | 4 |
| Information sources | 7 | PubMed, Embase and Web of Science were searched from the first available year to June 1, 2012, as well as hand searching of the references of identiﬁed articles were performed. | 3 |
| Search | 8 | Search strategy: “NADPH oxidase” AND “mutation OR variant OR polymorphism OR genotype” AND “stroke OR cerebrovascular disease OR cerebrovascular disorder OR cerebral infarction OR cerebral ischemia OR brain infarction”. | 3-4 |
| Study selection | 9 | Two investigators (Li BH and Zhang LL) screened each of the titles, abstracts, and full texts to determine inclusion independently. The results were compared and disagreements were resolved by consensus. | 4 |
| Data collection process | 10 | Information was carefully extracted from all included publications independently by two of the authors (Li BH and Zhang LL) according to the inclusion criteria listed above. Disagreement was resolved by consensus. If these two authors could not reach a consensus, another author (Li JC) was consulted. | 4 |
| Data items | 11 | The following data were collected from each study: first author’s name, publication date, country, ethnicity, study design (source of controls), phenotype (type of ICVD), diagnoses of ICVD (Clinical or imaging diagnosis), total numbers of cases and controls, frequency of C242T polymorphism in cases and controls or published crude odds ratios (ORs) derived from these data and evidence of HWE (P value less than 0.05 of HWE was considered significant), respectively. Different ethnicities were categorized as Caucasian, Asian, African and mixed. Study design was stratiﬁed to population-based (PB) studies and hospital-based (HB) studies. | 4 |
| Risk of bias in individual studies | 12 | The quality of included studies was evaluated independently by two authors (Li BH and Zhang LL) of this article according to the Newcastle-Ottawa Scale (NOS). | 4-5 |
| Summary measures | 13 | The principal summary measures are odds ratios (ORs) and 95% confidence intervals (CIs). | 5 |
| Synthesis of results | 14 | The strength of association between C242T polymorphism and ICVD risk was measured by Ors and 95%CIs. The combined ORs were calculated respectively for allelic model (T vs. C), additive model (TT vs. CC), dominant model (TT+TC vs. CC) and recessive model (TT vs. TC+CC). We used the generic inverse variance method to obtain pooled ORs, weighting each study by the inverse of the square of the standard error of its study-specific OR. The ORs were pooled through a fixed effects model when no heterogeneity was observed among studies. Otherwise, a random effects model was adopted. | 5 |

| **Section/topic** | **#** | **Checklist item** | **Page #** |
| --- | --- | --- | --- |
| Risk of bias across studies | 15 | An estimate of potential publication bias was carried out by Begg’s funnel plot and Egger’s regression test (p < 0.05 was considered representative of statistically signiﬁcant publication bias). | 5 |
| Additional analyses | 16 | Subgroup analyses were performed by ischemic stroke subtypes. Sensitivity analysis was performed by including studies of one ethnic group and by limiting the meta-analysis to studies in agreement with HWE. | 5 |
| **RESULTS** | | |  |
| Study selection | 17 | Based on our preliminary search criteria, a total of nine publications were eligible. Among these articles, one study was review article. Two studies reported the p47phox C923T rather than p22phox C242T polymorphism. Hence, six studies were included in the final meta-analysis, including 1,948 cases and 2,357 controls. | 6 |
| Study characteristics | 18 | Table 1 shows the studies included in the meta-analysis and their main characteristics. | 15 |
| Risk of bias within studies | 19 | The NOS results were shown in Table 1. The NOS results showed that the average score was 8.5 (range 8 to 9), indicating that the methodological quality was generally good. | 6 |
| Results of individual studies | 20 | The main results of individual studies were shown in Figure 2, respectively (Figure 2 Forest plots for overall studies). |  |
| Synthesis of results | 21 | There was no evidence of association between p22phox C242T polymorphism and overall ICVD (allelic model: OR=1.08, 95%CI=0.93-1.26; additive model: OR=1.33, 95%CI=0.81-2.17; dominant model: OR=1.00, 95%CI=0.86-1.15; and recessive model: OR=1.06, 95%CI=0.77-1.45). | 7 |
| Risk of bias across studies | 22 | The shapes of the funnel plots did not reveal any evidence of obvious asymmetry visually (Figure 3). Also there was no statistical evidence of publication bias among studies by using Egger’s regression test (P=0.28 for allelic model; P=0.38 for additive model; P=0.24 for dominant model and P=0.32 for recessive model, respectively). | 8 |
| Additional analysis | 23 | **Subgroup analysis:** When meta-analysis was performed to assess association between p22phox C242T polymorphism and small-vessel occlusive and large-artery atherosclerotic stroke, statistical significant association was found in recessive model demonstrating that TT genotype could increase the risk of large-artery atherosclerotic stroke of 2.17 fold (allelic model: OR=1.12, 95%CI=0.88-1.41; additive model: OR=1.36, 95%CI=0.60-3.09; dominant model: OR=1.25, 95%CI=0.74-2.11; and recessive model: OR=2.17, 95%CI=1.11-4.23), whereas no evidence for significant association was observed for small-vessel occlusive stroke (allelic model: OR=1.16, 95%CI=0.94-1.44; additive model: OR=1.32, 95%CI=0.28-6.12; dominant model: OR=1.11, 95%CI=0.84-1.47; recessive model: OR=0.90, 95%CI=0.62-1.31). No statistical evidence for significant association was observed for Asian subgroup and Caucasian subgroup. Statistical powers on the combined sample size (total and subgroup) were all power than 80%. | 7-8 |
| **DISCUSSION** | | |  |
| Summary of evidence | 24 | A total of six publications were included in our meta-analysis. Our meta-analyses did not show statistical evidence for association between the NADPH oxidase p22phox C242T polymorphism and ICVD in the overall study population. | 8-9 |
| Limitations | 25 | **Limitations:** Firstly, the small number of studies and sample size limited the ability to draw more solid conclusions. Secondly, lacking of the original data limited our further evaluation of potential interactions among gene-gene and gene-environment. | 8 |
| Conclusions | 26 | **Conclusion：**Our meta-analysis suggests that C242T polymorphism is more associated with large-artery atherosclerotic stroke than small-vessel occlusive stroke. However, this conclusion should be interpreted with caution due to the small sample size. Larger sample-size studies with homogeneous ICVD patients and well-matched controls are required. | 10 |
| **FUNDING** | | |  |
| Funding | 27 | This study was supported by grants from the National Natural Science Foundation of China (81271282), Chongqing Natural Science Foundation (CSTC2011BB5031). | 11 |

*From:*  Moher D, Liberati A, Tetzlaff J, Altman DG, The PRISMA Group (2009). Preferred Reporting Items for Systematic Reviews and Meta-Analyses: The PRISMA Statement. PLoS Med 6(6): e1000097. doi:10.1371/journal.pmed1000097

For more information, visit: **www.prisma-statement.org**.

Page 2 of 2
