# Supplementary material for: Association between NADPH Oxidase p22phox C242T Polymorphism and Ischemic Cerebrovascular Disease: A Meta-Analysis
Source: PLoS One. 2013 Feb 11;8(2):e56478. doi: 10.1371/journal.pone.0056478 (PMC3569432; doi:10.1371/journal.pone.0056478)
Supplement: Appendix S3 — MOOSE Checklist. (DOC) [file pone.0056478.s003.doc]

**MOOSE Checklist**

| **Criteria** | | **Brief description of how the criteria were handled in the meta-analysis** |
| --- | --- | --- |
| **Reporting of background should include** | |  |
| √ | Problem definition | NADPH oxidase p22phox C242T polymorphism is closely associated with various diseases including renal disease, hypertension, diabetes, cardiovascular disease and cerebrovascular disease. The association between C242T polymorphism and susceptibility to ischemic cerebrovascular disease were inconsistent in previous studies. |
| √ | Hypothesis statement | It is likely that NADPH oxidase p22phox C242T polymorphism may influence the susceptibility of ICVD. |
| √ | Description of study outcomes | Ischemic cerebrovascular disease |
| √ | Type of exposure or intervention used | TC, CC, TC+CC genotypes or T allele |
| √ | Type of study designs used | Published case-control, nested case-control or cohort designs studies. |
| √ | Study population | No restriction. |
| **Reporting of search strategy should include** | |  |
| √ | Qualifications of searchers | Investigators include experts in cerebrovascular disease and qualified graduate students. All of the investigators have received training in literature research, statistics and evidence-based medicine. |
| √ | Search strategy, including time period included in the synthesis and keywords | We selected possibly relevant articles in Embase (1966-June 2012), PubMed (up to June 2012) and Web of Science (1950-June 2012) (last search was update on June 1, 2012) with search strategy: “NADPH oxidase” AND “mutation OR variant OR polymorphism OR genotype” AND “stroke OR cerebrovascular disease OR cerebrovascular disorder OR cerebral infarction OR cerebral ischemia OR brain infarction”. |
| √ | Databases and registries searched | PubMed, EMBASE and Web of Science |
| √ | Search software used, name and version, including special features | We did not employ any search software. EndNote was used to merge retrieved citations and eliminate duplications. |
|  | Use of hand searching | Other relevant studies were identiﬁed by hand-searching the references of included articles identiﬁed by electronic search and the abstracts presented at related scientific societies meetings. |
| √ | List of citations located and those excluded, including justifications | Literature search and selection process are outlined in the flow diagram. The reasons for exclusion were listed in the flow diagram and explained in result section. |
|  | Method of addressing articles published in languages other than English | The search was limited to English and Chinese language papers. |
| √ | Method of handling abstracts and unpublished studies | We first examined if overlap existed and excluded overlapped studies. We only included published case-control, nested case-control or cohort designs studies. |
| √ | Description of any contact with authors | If necessary data were not reported in the primary manuscripts, we contacted the corresponding authors by email to request the missing data. |
| **Reporting of methods should include** | |  |
| √ | Description of relevance or appropriateness of studies assembled for assessing the hypothesis to be tested | Detailed inclusion and exclusion criteria were described in the methods. |
| √ | Rationale for the selection and coding of data | We only used the crude ORs and 95%CIs for meta-analysis. If the studies did not provide crude ORs and 95%CIs, we calculated the ORs and 95%CIs by the total numbers of cases and controls, and frequency of C242T polymorphism in cases and controls. |
| √ | Assessment of confounding | NOS rating system was used to assess the confounder. Subgroup analysis of stroke subtype was performed and sensitivity analyses were also performed. |
| √ | Assessment of study quality, including blinding of quality assessors; stratification or regression on possible predictors of study results | We assessed the methodological qualities of included studies by the description of study population, the set of controls and cases and related statistical methods. We carried out sensitivity analysis. |
| √ | Assessment of heterogeneity | Between-study heterogeneity was assessed by the Q-test and I2 statistic, P＜0.10 and I^2^>50% indicated evidence of heterogeneity. |
| √ | Description of statistical methods in sufficient detail to be replicated | Methods of heterogeneity test, quantitative synthesis, assessments of publication bias, sensitivity analyses are reported in detail in the methods section. |
| √ | Provision of appropriate tables and graphics | We provided flow chart to explain literature searching and selection (Figure 1); forest plots for the total analysis, subgroup analyses and sensitivity analysis (Figure 2, Appendix S4, Appendix S5, Appendix S6, Appendix S7); study characteristics and allele/genotype frequencies (Table 1). |
| **Reporting of results should include** | |  |
|  | Graph summarizing individual study estimates and overall estimate | Graph summarizing individual study estimates and overall estimate are presenting in Figure 2. |
| √ | Table giving descriptive information for each study included | Descriptive information for each study included was provided in Table 1. |
| √ | Results of sensitivity testing | The results of sensitivity analysis were described in results section. Appendix S6 and S7 provide detailed forest plot for the sensitivity analysis of Asian population and Caucasian population. |
| √ | Indication of statistical uncertainty of findings | The results of heterogeneity test, pooled ORs, 95% confidence intervals and *P* value for *Z* test were presented with all pooled analyses. Power calculations were performed on the given sample size. |
| **Reporting of discussion should include** | |  |
| √ | Quantitative assessment of bias | We evaluated the publication bias by funnel plots and egger’s test. No significant publication bias was detected. |
| √ | Justification for exclusion | Based on our preliminary search criteria, a total of nine publications were eligible. Among these articles, one study was review article. Two studies reported the p47phox C923T rather than p22phox C242T polymorphism. |
| √ | Assessment of quality of included studies | We discussed the results of sensitivity analysis and described the limitations of included studies. |
| **Reporting of conclusions should include** | |  |
| √ | Consideration of alternative explanations for observed results | We discussed that potential unmeasured confounders and explained the limitations of this meta-analysis. We reminded readers that caution should be made when interpreting this meta-analysis. |
| √ | Generalization of the conclusions | Our meta-analysis suggests that C242T polymorphism is more associated with large-artery atherosclerotic stroke than small-vessel occlusive stroke. |
| √ | Guidelines for future research | Larger sample-size studies with homogeneous ICVD patients and well-matched controls are required. |
| √ | Disclosure of funding source | This study was supported by grants from the National Natural Science Foundation of China (81271282), Chongqing Natural Science Foundation (CSTC2011BB5031). |
