# Supplementary figures and images for: Association between NADPH Oxidase p22phox C242T Polymorphism and Ischemic Cerebrovascular Disease: A Meta-Analysis
Source: PLoS One. 2013 Feb 11;8(2):e56478. doi: 10.1371/journal.pone.0056478 (PMC3569432; doi:10.1371/journal.pone.0056478)

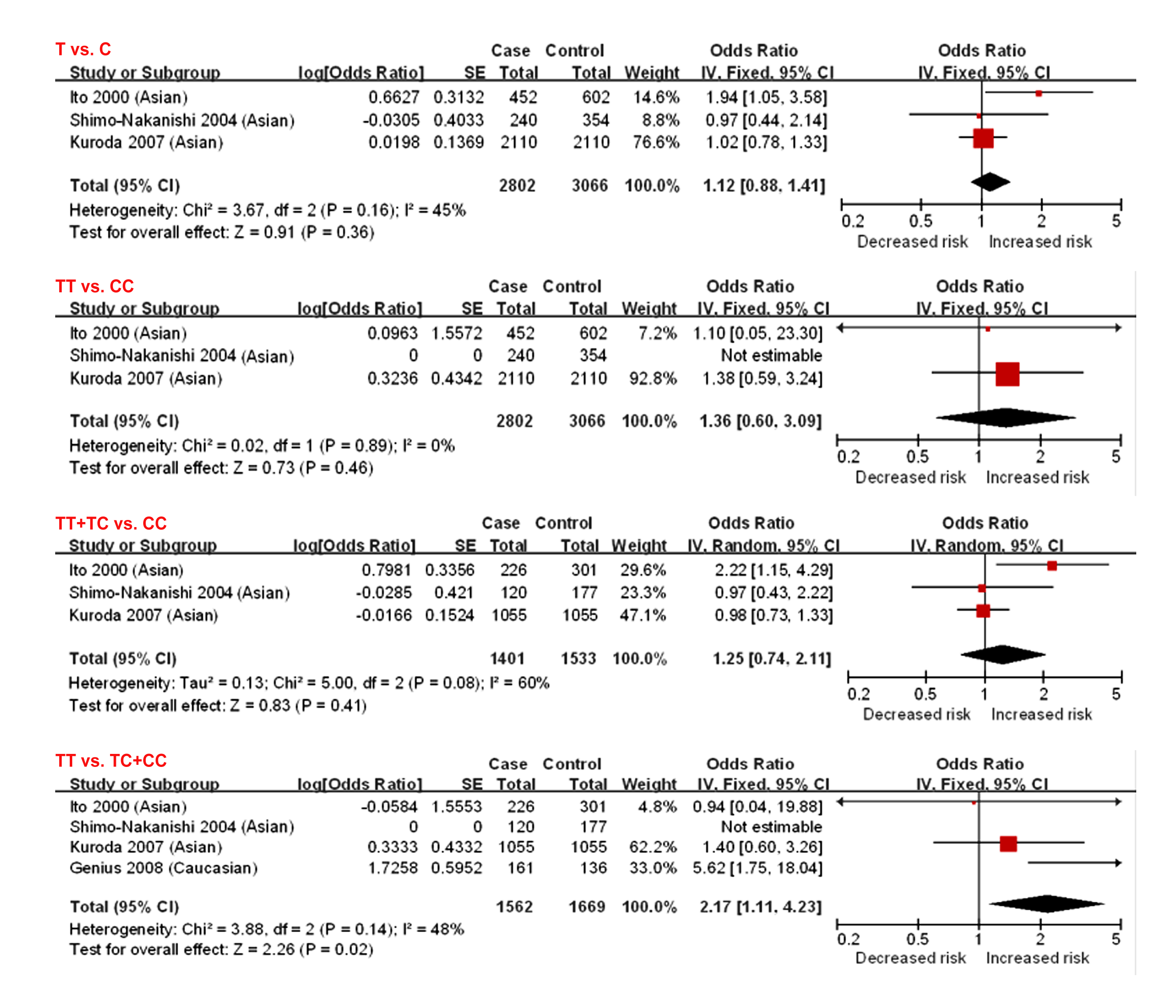

Supplement: Appendix S4 — Forest plots for large-artery atherosclerotic subtype. (TIF) [file pone.0056478.s004.tif]

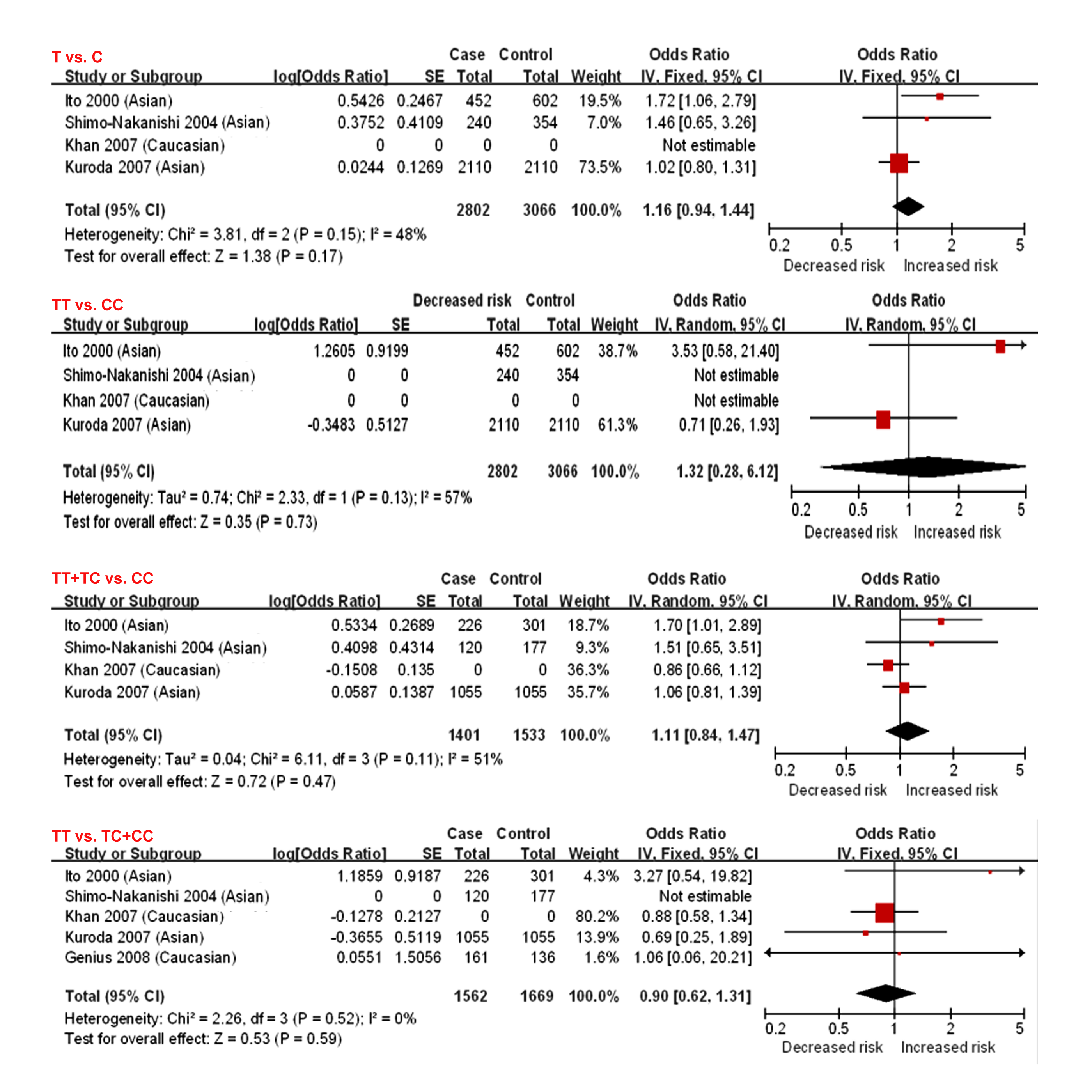

Supplement: Appendix S5 — Forest plots for small-vessel occlusive subtype. (TIF) [file pone.0056478.s005.tif]

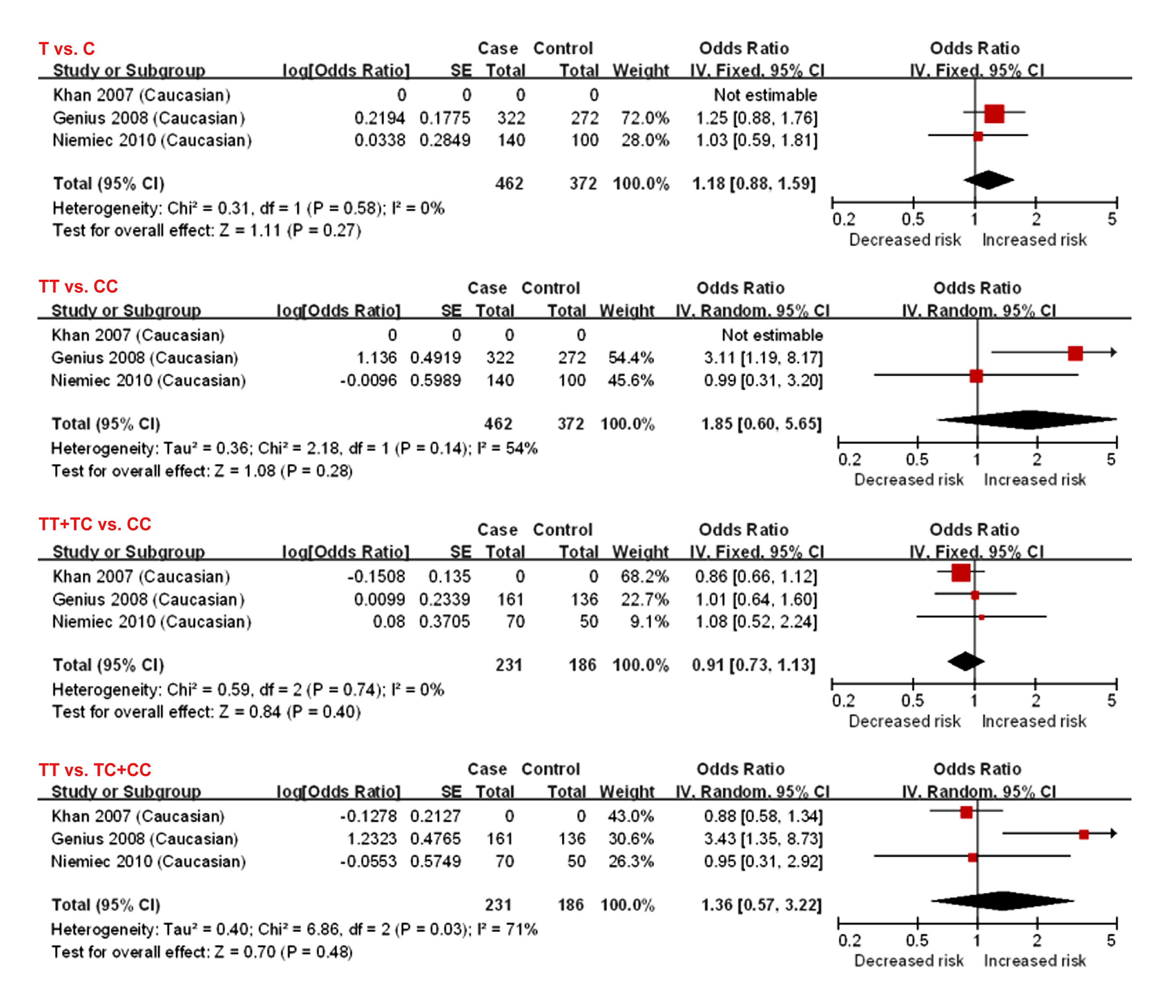

Supplement: Appendix S6 — Forest plots for Caucasian subgroup. (TIF) [file pone.0056478.s006.tif]

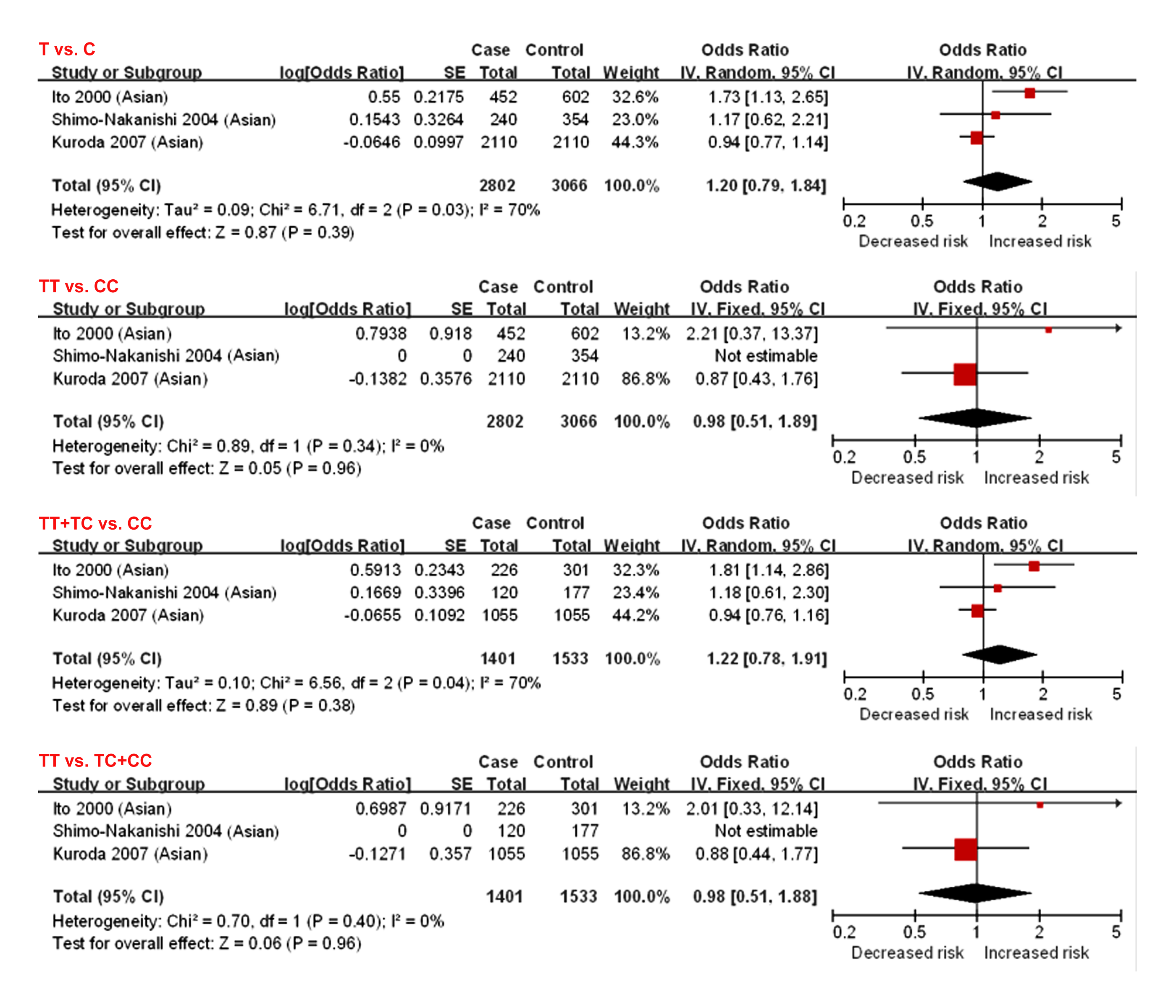

Supplement: Appendix S7 — Forest plots for Asian subgroup. (TIF) [file pone.0056478.s007.tif]
